# Supplementary material for: Systematic analysis of ChatGPT, Google search and Llama 2 for clinical decision support tasks
Source: Nat Commun. 2024 Mar 6;15:2050. doi: 10.1038/s41467-024-46411-8 (PMC10917796; doi:10.1038/s41467-024-46411-8)
Supplement: Supplementary file 1 — Supplementary Information [file 41467_2024_46411_MOESM1_ESM.pdf]

Progression of Large Language Models for Clinical Decision Support – Comparing GPT, Llama and Google Search

Table of Contents

1. Supplementary Methods 2
2. Supplementary Figures 4
3. Supplementary Tables 10

1. Supplementary Methods

In our study, we aim at analyzing the influence of a diseases' incidence (rare vs less frequent vs frequent) on the performance of GPT-3.5, GPT-4 and Google. To estimate the power in relation to sample size, we make the following assumptions:

- The performance of GPT-4 is better compared to GPT-3.5.
- The performance of GPT-4 and GPT-3.5 is better compared to Google.
- The performance of all approaches shows a decrease in performance with decreasing frequency of the disease.

The following seven tests will be applied on all three levels diagnosis, examination and treatment:

1. One-sided non-paired Mann-Whitney test comparing Likert scale rating for frequent vs less frequent diseases, using GPT-3.5.
2. One-sided non-paired Mann-Whitney test comparing Likert scale rating for frequent vs rare diseases, using GPT-3.5.
3. One-sided non-paired Mann-Whitney test comparing Likert scale rating for less frequent vs rare diseases, using GPT-3.5.
4. One-sided non-paired Mann-Whitney test comparing Likert scale rating for frequent vs less frequent diseases, using GPT-4.
5. One-sided non-paired Mann-Whitney test comparing Likert scale rating for frequent vs rare diseases, using GPT-4.
6. One-sided non-paired Mann-Whitney test comparing Likert scale rating for less frequent vs rare diseases, using GPT-4.
7. One-sided paired Mann-Whitney test comparing Likert scale rating for GPT-3.5 vs GPT-4.

In order to investigate the effect on diagnosis, five additional tests will be applied:

8. One-sided non-paired Mann-Whitney test comparing Likert scale rating for frequent vs less frequent diseases, using Google.
9. One-sided non-paired Mann-Whitney test comparing Likert scale rating for frequent vs rare diseases, using Google.
10. One-sided non-paired Mann-Whitney test comparing Likert scale rating for less frequent vs rare diseases, using Google.
11. One-sided paired Mann-Whitney test comparing Likert scale rating for GPT-3.5 vs Google.
12. One-sided paired Mann-Whitney test comparing Likert scale rating for GPT-4 vs Google.

Using R 4.3.1, we sample data with the following probabilities for Likert scale ratings 1 / 2 / 3 / 4 / 5:

- GPT-3.5, frequent: 0.00 / 0.075 / 0.175 / 0.50 / 0.25
- GPT-3.5, less frequent: 0.075 / 0.175 / 0.50 / 0.175 / 0.075
- GPT-3.5, rare: 0.25 / 0.50 / 0.175 / 0.075 / 0.00
- GPT-4, frequent: 0.00 / 0.00 / 0.075 / 0.25 / 0.675
- GPT-4, less frequent: 0.00 / 0.075 / 0.175 / 0.50 / 0.25

- GPT-4, rare: 0.075 / 0.175 / 0.50 / 0.175 / 0.075
- Google, frequent: 0.075 / 0.175 / 0.50 / 0.175 / 0.075
- Google, less frequent: 0.25 / 0.50 / 0.175 / 0.075 / 0.00
- Google, rare: 0.675 / 0.25 / 0.075 / 0.00 / 0.00

The R-script that is executed to estimate the power with sample sizes between 20 and 45, considering diagnosis, examination and treatment, is provided as Supplementary Data 4.

The results are summed up in Supplementary Figure 1.

Based on our simulation, a sample size of 33 per frequency group leads to a power of 0.9035 when adjusting for 7 tests. A sample size of 38 to a power of 0.9055 when performing 12 tests. Thus, to achieve sufficient power on all three levels – diagnosis, examination and treatment – on one side, without over-powering the study on the other side, we aimed for 33-38 cases per frequency group.

2. Supplementary Figures

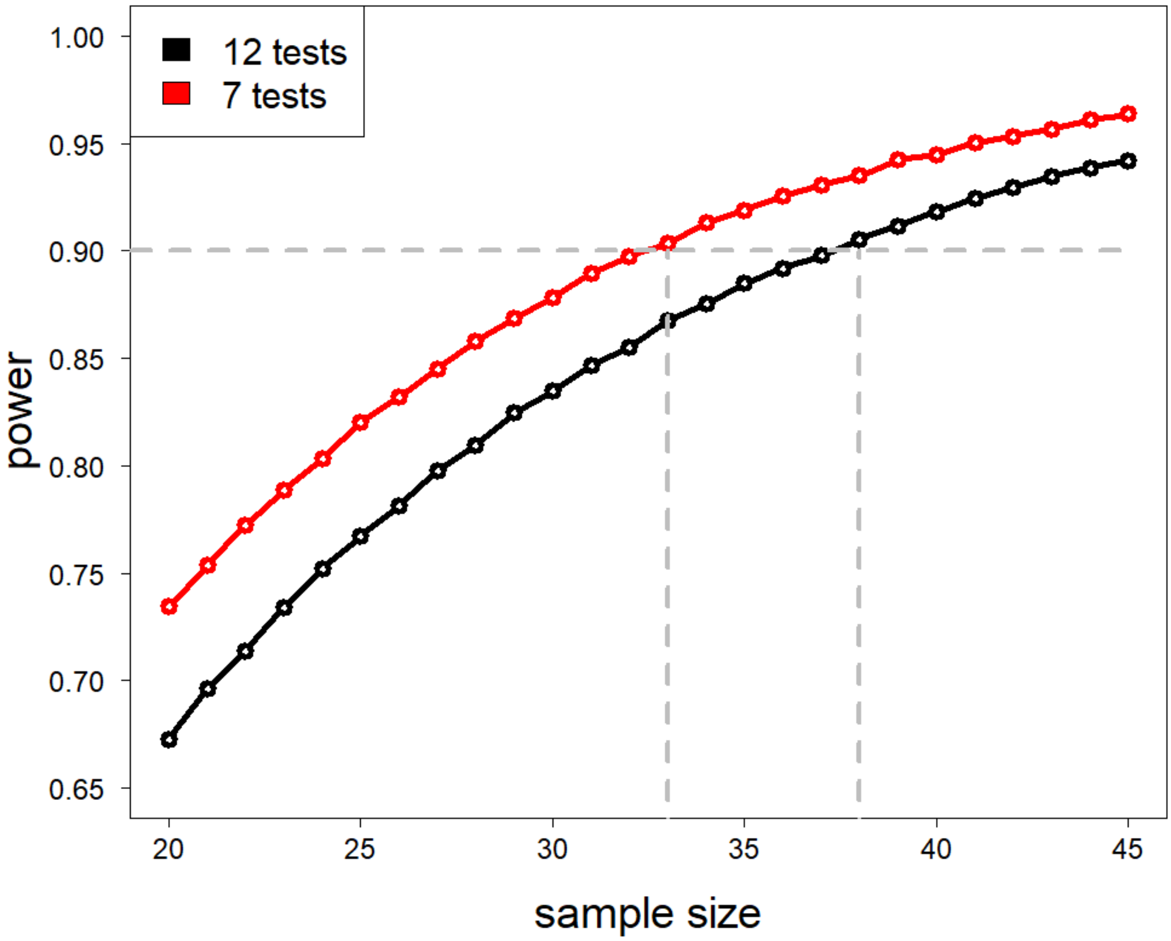

Supplementary Figure 1. Sample size estimation. Sample size in relation to estimated power when performing 12 and 7 tests.

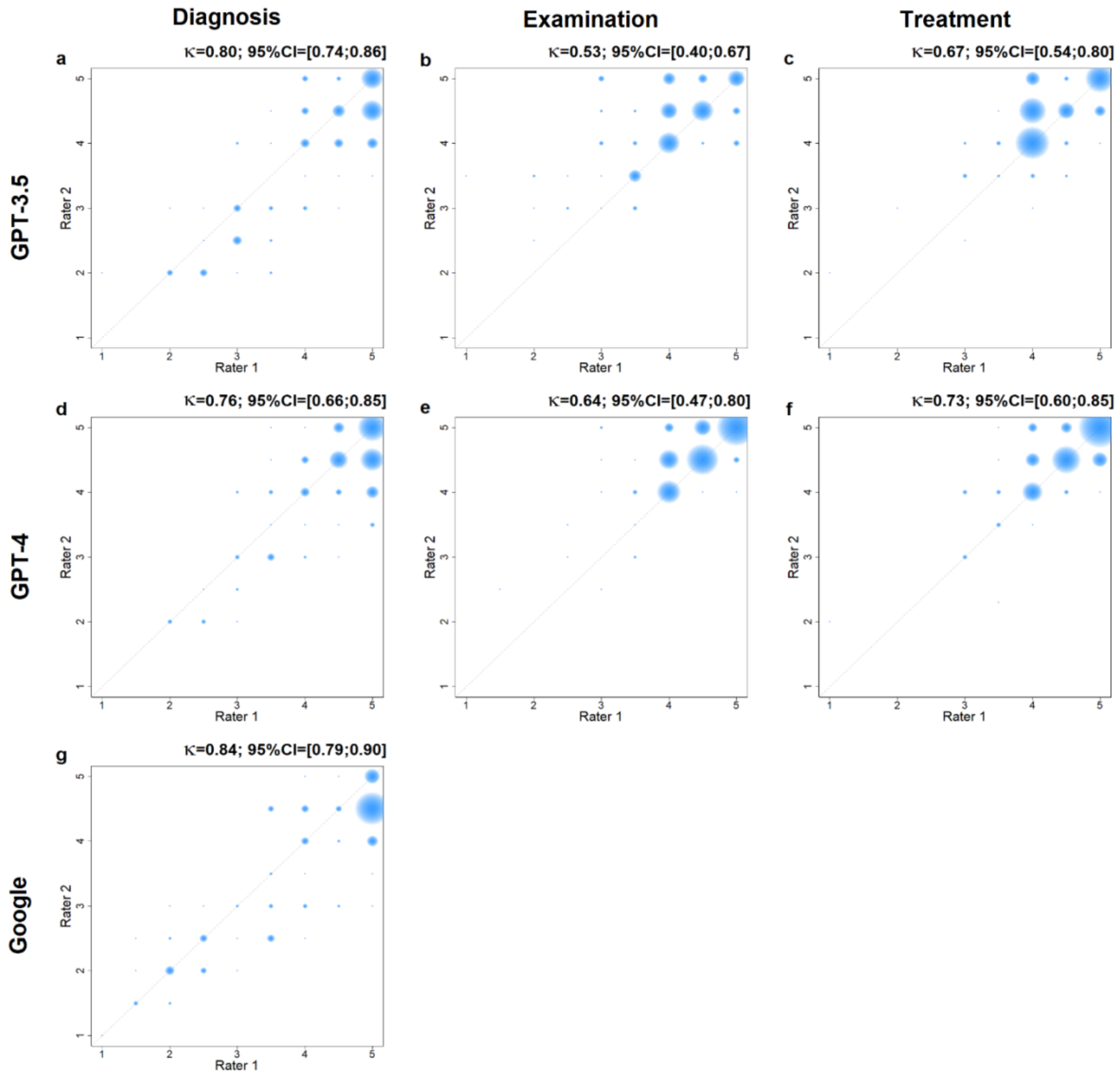

Supplementary Figure 2. Inter-rater reliability considering a 5-point Likert scale. The performance of **a** GPT-3.5 diagnosis; **b** GPT-3.5 examination; **c** GPT-3.5 treatment; **d** GPT-4 diagnosis; **e** GPT-4 examination; **f** GPT-4 treatment, **g** Google diagnosis is compared on all *n*=110 cases.

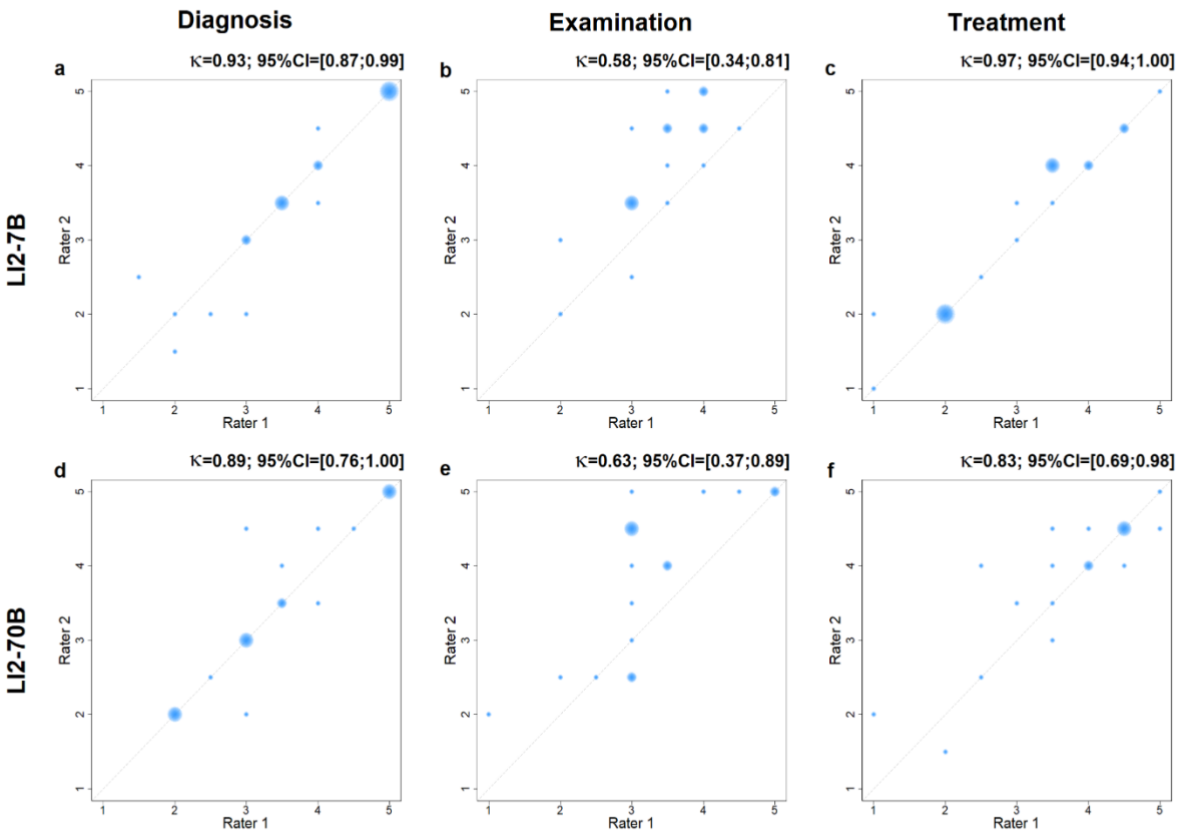

Supplementary Figure 3. Inter-rater reliability considering a 5-point Likert scale. The performance of **a** Ll2-7B diagnosis; **b** Ll2-7B examination; **c** Ll2-7B treatment; **d** Ll2-70B diagnosis; **e** Ll2-70B examination; **f** Ll2-70B diagnosis is compared on a subset of *n*=18 cases. Ll2-7B: Llama-2-7b-chat; Ll2-70B: Llama-2-70b-chat.

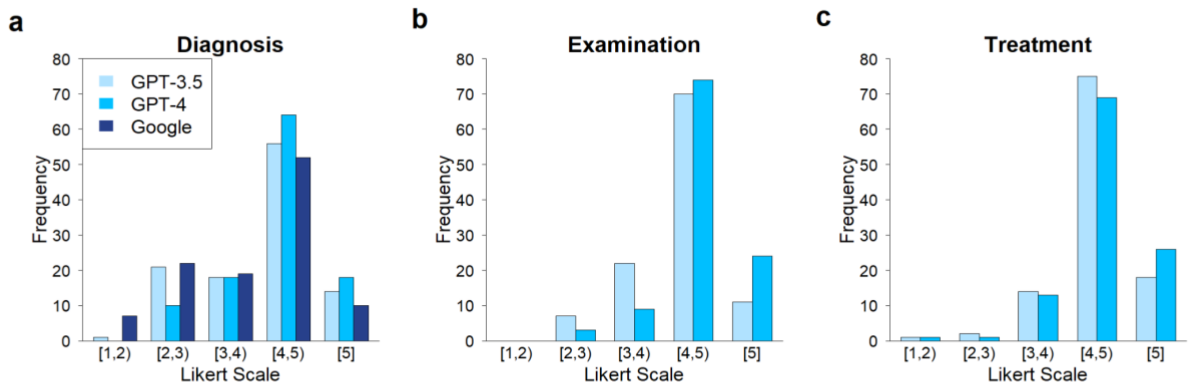

Supplementary Figure 4. Summarized performance per task for the three approaches. Performance of GPT-3.5, GPT-4 and Google considering **a** diagnosis, **b** examination and **c** treatment.

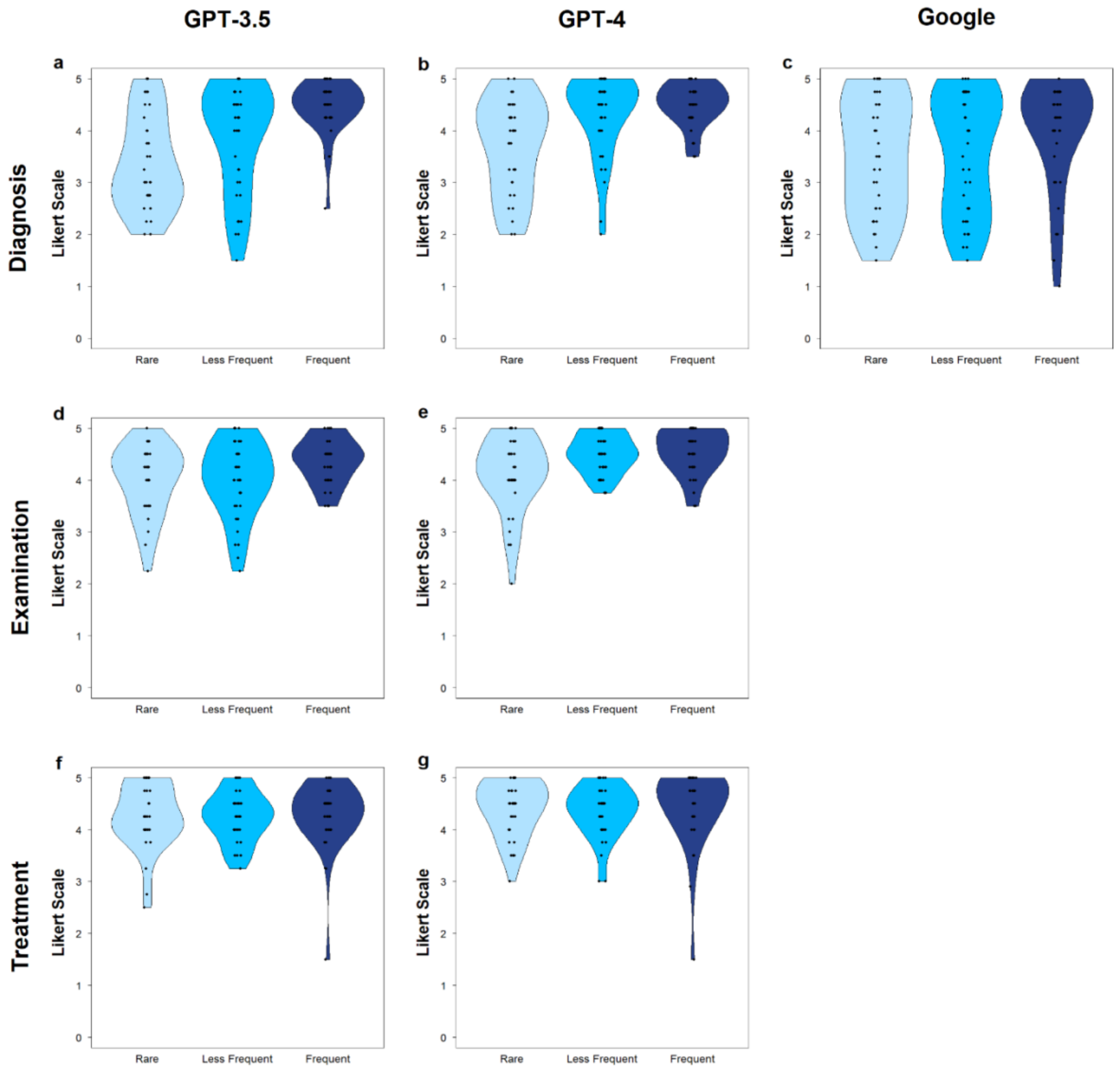

Supplementary Figure 5. Detailed performance per task for GPT-3.5, GPT-4 and Google. Performance of **a** GPT-3.5, **b** GPT-4 and **c** Google considering diagnosis. Performance of **d** GPT-3.5 and **e** GPT-4 considering examination. Performance of **f** GPT-3.5 and **g** GPT-4 considering treatment. Violin plots show the performance of each tool in relation to disease frequency.

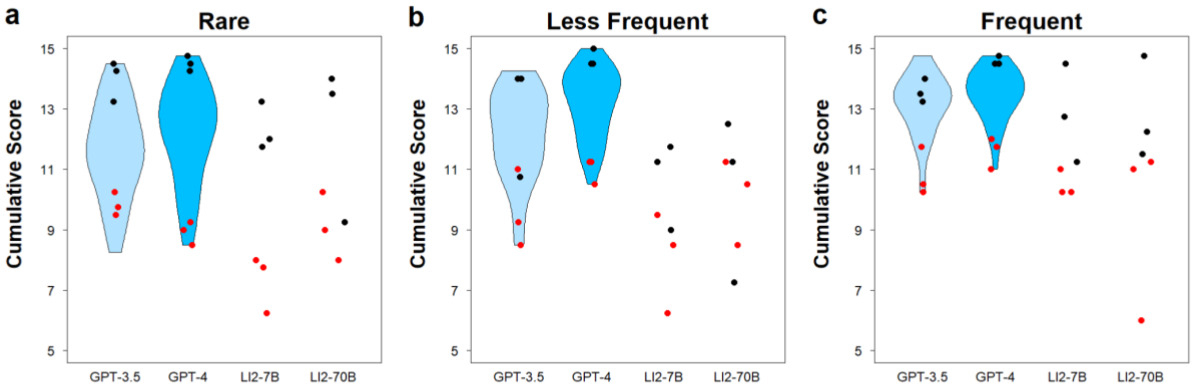

Supplementary Figure 6: **Performance per frequency category comparing GPT-3·5 vs GPT-4 vs Ll2-7B vs Ll2-70B**. Performance of GPT-3·5 and GPT-4 vs Ll2-7B and Ll2-70B considering A) rare, B) less frequent and C) frequent diseases. Violin plots visualize the performance of GPT-3·5 and GPT-4. Black dots mark the top-3 cases based on GPT-4's cumulative score for rare, less frequent and frequent diseases. Red dots the bottom-3 cases. Ll2-7B: Llama-2-7b-chat; Ll2-70B: Llama-2-70b-chat.

3. Supplementary Tables

Supplementary Table 1. Performance per task and per frequency category for GPT-3.5, GPT-4 and Google. Performance of GPT-3.5, GPT-4 and Google measured by median score and inter-quartile range, considering diagnosis, examination and treatment for all, rare, less frequent and frequent diseases.

	**All**	**Rare**	**Less Frequent**	**Frequent**
Diagnosis				
➢ GPT-3.5 [median; IQR]	4.25 [3.00;4.75]	3.00 [2.75;4.00]	4.25 [3.13;4.63]	4.50 [4.25;4.75]
➢ GPT-4 [median; IQR]	4.50 [3.81;4.75]	4.00 [3.00;4.50]	4.50 [4.00;4.75]	4.50 [4.25;4.75]
➢ Google [median; IQR]	4.00 [2.75;4.75]	3.50 [2.50;4.50]	4.00 [2.50;4.75]	4.25 [3.75;4.75]
Examination				
➢ GPT-3.5 [median; IQR]	4.25 [3.75;4.50]	4.25 [3.50;4.50]	4.00 [3.63;4.50]	4.50 [4.00;4.50]
➢ GPT-4 [median; IQR]	4.50 [4.00;4.75]	4.13 [4.00;4.50]	4.50 [4.25;4.75]	4.50 [4.25;5.00]
Treatment				
➢ GPT-3.5 [median; IQR]	4.25 [4.00;4.69]	4.25 [4.00;4.75]	4.25 [4.00;4.50]	4.50 [4.00;4.75]
➢ GPT-4 [median; IQR]	4.50 [4.00;4.75]	4.50 [4.00;4.75]	4.50 [4.00;4.75]	4.75 [4.25;5.00]

Supplementary Table 2. Results of statistical tests per task and per frequency category for GPT-3.5, GPT-4 and Google. *P* values and adjusted *p* values evaluating the performance of GPT-3.5, GPT-4 and Google considering diagnosis, examination and treatment for all, rare, less frequent and frequent diseases. One-sided Mann-Whitney test was applied for statistical testing (adjusted with Bonferroni correction for multiple testing considering n=12 tests for diagnosis, n=7 tests for examination and treatment). Exact not-adjusted *p* values are: for diagnosis Google vs GPT-4 all $p=4.6769*10^{-5}$; for diagnosis GPT-3.5 rare vs frequent $p=1.1464*10^{-6}$; for diagnosis GPT-4 rare vs frequent $p=2.8784*10^{-5}$; for examination GPT-3.5 vs GPT-4 all $p=4.6058*10^{-7}$. Exact adjusted *p* values are: for diagnosis GPT-3.5 rare vs frequent *p* adj=$1.3757*10^{-5}$; for examination GPT-3.5 vs GPT-4 all p adj=$3.2241*10^{-6}$.

		**x**	**y**	***p***	***p* adj**
Diagnosis	All	GPT-3.5	GPT-4	0.0003	**0.0033**
		Google	GPT-3.5	0.0518	0.6215
		Google	GPT-4	<0.0001	**0.0006**
	GPT-3.5	Rare	Less frequent	0.0121	0.1447
		Rare	Frequent	<0.0001	**<0.0001**
		Less frequent	Frequent	0.0074	0.0890
	GPT-4	Rare	Less frequent	0.0006	**0.0067**
		Rare	Frequent	<0.0001	**0.0003**
		Less frequent	Frequent	0.3835	1
	Google	Rare	Less frequent	0.4779	1
		Rare	Frequent	0.0624	0.7492
		Less frequent	Frequent	0.0904	1
Examination	All	GPT-3.5	GPT-4	<0.0001	**<0.0001**
	GPT-3.5	Rare	Less frequent	0.4844	1
		Rare	Frequent	0.0095	0.0665
		Less frequent	Frequent	0.0133	0.0928
	GPT-4	Rare	Less frequent	0.0082	0.0572
		Rare	Frequent	0.0029	**0.0203**
		Less frequent	Frequent	0.2398	1
Treatment	All	GPT-3.5	GPT-4	0.0072	0.0503
	GPT-3.5	Rare	Less frequent	0.5844	1
		Rare	Frequent	0.3054	1
		Less frequent	Frequent	0.1566	1
	GPT-4	Rare	Less frequent	0.6858	1
		Rare	Frequent	0.1912	1
		Less frequent	Frequent	0.0724	0.5066
