# Supplementary material for: Systematic analysis of ChatGPT, Google search and Llama 2 for clinical decision support tasks
Source: Nat Commun. 2024 Mar 6;15:2050. doi: 10.1038/s41467-024-46411-8 (PMC10917796; doi:10.1038/s41467-024-46411-8)
Supplement: Supplementary file 3 — Description of Additional Supplementary Files [file 41467_2024_46411_MOESM3_ESM.pdf]

Description of Additional Supplementary Files

Supplementary Data 1: Overview of all clinical cases their source information and assessment.

Supplementary Data 2: R script generating Figure 1 of the main manuscript.

Supplementary Data 3: R script generating Figure 2 of the main manuscript.

Supplementary Data 4: R script performing power analysis as described in Supplementary Methods.
